# A Practical Protocol for Reporting Depressive Symptoms in a Clinical Trial of Grandmother Caregivers

**DOI:** 10.1093/geroni/igaa057.537

**Published:** 2020-12-16

**Authors:** McKenzie Wallace, Alexandra Jeanblanc, Carol Musil

**Affiliations:** 1 Case Western Reserve University, Lorain, Ohio, United States; 2 Case Western Reserve University, Cleveland, Ohio, United States

## Abstract

Intervention studies conducted in caregivers often focus on improving mental health. Consequently, researchers may discover incidental findings like elevated depressive symptoms. Researchers have an ethical obligation to report incidental findings to participants, but no protocols exist for reporting behavioral health symptoms. This presentation describes a protocol for reporting elevated depressive symptoms to participants, based on the protocol used in a national randomized clinical trial of stress-reduction methods for 348 grandmothers raising grandchildren. Each questionnaire included the CES-D scale and was scored immediately after completion. Based on our previous work showing higher CES-D scores in custodial grandmothers, and the desire to balance the principles of beneficence and nonmaleficence, we established a cut-off score of 30. A registered nurse on the research team called participants with scores over 30 and ascertained whether the participant 1) was aware of the problem and 2) had sought help, and then offered additional resources. Overall, 94 (27%) participants had a CES-D score > 30. The majority (91%) were aware of the problem. About a third of the participants were on medication for their symptoms, and a third were seeing a therapist. Nine participants were not aware they had depressive symptoms. While most of our participants were aware of their depressive symptoms, they were appreciative of our call and several noted that they would speak to their provider. Future studies should consider how to implement a similar protocol to deliver information critical to the mental health of participants.

